# The synovial fluid proteome differentiates between septic and nonseptic articular pathologies

**DOI:** 10.1016/j.jprot.2019.04.020

**Published:** 2019-06-30

**Authors:** James R. Anderson, Aibek Smagul, Deborah Simpson, Peter D. Clegg, Luis M. Rubio-Martinez, Mandy J. Peffers

**Affiliations:** a Institute of Ageing and Chronic Disease, William Henry Duncan Building, 6 West Derby Street, Liverpool L7 8TX, UK; b Centre for Proteome Research, Institute of Integrative Biology, Biosciences Building, Crown Street, University of Liverpool, Liverpool L69 7ZB, UK; c Department of Equine Clinical Studies, Institute of Veterinary Science, Chester High Road, Neston CH64 7TE, UK

**Keywords:** Synovial fluid, Arthropathy, Osteochondrosis, Osteoarthritis, Synovial sepsis,110 equine

## Abstract

Articular conditions are common in horses and can result in loss of function, chronic pain and/or inability to work. Common conditions include osteoarthritis, osteochondrosis and synovial sepsis, which can be life-threatening, but despite the high clinical prevalence of these conditions, rapid and specific diagnosis, monitoring and prognostication remains a challenge for practicing veterinarians. Synovial fluid from a range of arthropathies was enriched for low abundance proteins using combinatorial peptide ligand ProteoMiner™ beads and analysed via liquid chromatography-tandem mass spectrometry. Changes in protein abundances were analysed using label-free quantification. Principle component analysis of differentially expressed proteins identified groupings associated with joint pathology. Findings were validated using ELISA. Lactotransferrin (LTF) abundance was increased in sepsis compared to all other groups and insulin-like growth factor-binding protein 6 (IGFBP6) abundance decreased in sepsis compared to other disease groups. Pathway analysis identified upregulation of the complement system in synovial joint sepsis and the downregulation of eukaryotic translation initiation factors and mTOR signalling pathways in both OA and OC compared to the healthy group. Overall, we have identified a catalogue of proteins which we propose to be involved in osteoarthritis, osteochondrosis and synovial sepsis pathogenesis.

**Significance:**

Osteoarthritis, osteochondrosis and synovial sepsis, which can be life-threatening, are common articular conditions in which rapid and specific diagnosis, monitoring and prognostication remains a challenge for practicing veterinarians. This study has identified that the equine synovial fluid proteome exhibits distinctive profile changes between osteoarthritis, osteochondrosis, synovial sepsis and healthy joints. Elevated synovial abundance of lactotransferrin and decreased insulin-like growth factor-binding protein 6 were both found to distinguish synovial sepsis from all other study groups. Thus, these protein markers may have a future role in clinical practice to enable an earlier and reliable diagnosis of synovial sepsis.

## Highlights

• Synovial fluid proteomics discriminated healthy from osteoarthritis, osteochondrosis and sepsis.
• Increased abundance of lactotransferrin in septic synovial fluid.
• Reduced abundance of insulin-like growth factor-binding protein 6 in septic synovial fluid.
• Sepsis upregulates coagulation, acute phase response signalling and complement systems.
• NFE2L2 was identified as the most significantly inhibited upstream regulator in osteochondrosis.

## Graphical abstract

**Figure:**
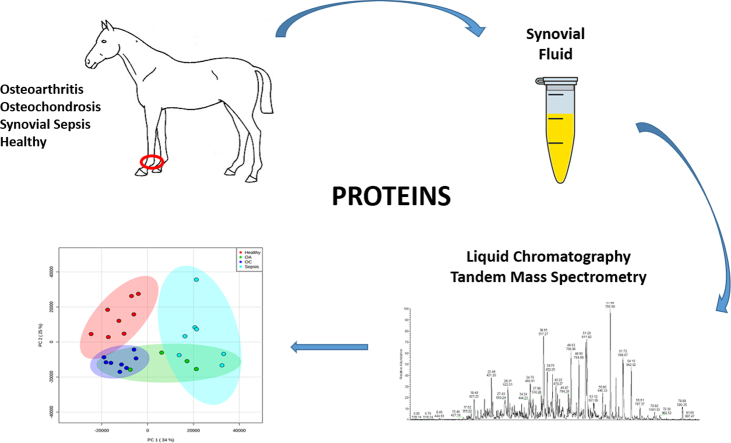

## 1 Introduction

Articular conditions are common in horses and can result in loss of function, chronic pain and/or inability to work, which are important welfare concerns. The most common conditions include osteoarthritis (OA), osteochondrosis (OC) and synovial sepsis, which can be life-threatening [1]. Despite the high clinical prevalence of these conditions, rapid and specific diagnosis, monitoring and prognostication remains a challenge for practicing veterinarians, thus, identification of reliable biomarkers of disease is required. Synovial fluid (SF) is in direct contact with articular structures and represents an important source of biomarker discovery. SF is located within the articular joint cavity, providing a pool of nutrients for surrounding tissues but primarily serving as a biological lubricant, containing molecules including hyaluronan with low-friction and low-wear properties to articular surfaces [2]. As SF is in close proximity to articular tissues primarily altered during joint pathology, this biofluid is an important first approach source for biomarker discovery [3,4].

Total protein content in synovial fluid is associated with presence of articular pathology and is one of the parameters most commonly used in equine clinical practice to enable diagnosis and to monitor horses with synovial sepsis [5]. Problematically there are large overlaps in the ranges of SF total protein content among arthropathies, and total protein content is also affected by articular diagnostic procedures and treatments [6]. This situation can lead to erroneous interpretations and clinical decisions, representing an important welfare risk to the horse. More specific proteins such as the acute phase inflammatory protein serum amyloid A has been investigated as a potential biomarker of articular conditions in horses [7]; however, it is easily affected by the systemic condition of the patient, and therefore, can be unreliable for specific clinical diagnosis [8].

Proteomics encompasses the comprehensive profiling of protein contents. The proteomic profile of the synovial fluid is dependent upon articular disease and its characterization has allowed the differentiation of OA and rheumatoid arthritis (RA) in man [4]. A recent study has identified a subset of proteins differentially expressed in OA versus normal equine joints [9]. Although an increase in total protein is a well-known feature of synovial sepsis, the protein profile in synovial sepsis has not been investigated. Identification of a characteristic proteomic profile in synovial sepsis would increase the understanding of the molecular basis of the condition and also enable the identification of potential biomarkers for early diagnosis and treatment of this disease.

A previous study has investigated the equine SF profile in OA and OC compared to healthy donors using 2D-gel electrophoresis with subsequent mass spectrometry analysis of in-gel tryptic digests [46]. However, few studies have investigated the proteomic profile of equine SF using high mass resolution mass spectrometer of in-solution tryptic digestions [9,10] and, to our knowledge, none have investigated protein profile changes in septic SF in any species. In this study we have used mass spectrometry and label-free quantification to identify potential protein biomarkers that allow differentiation between equine articular joint pathologies, potentially aiding accurate diagnosis and enabling increased understanding of the effect of septic synovitis on the joint environment. Identification of characteristic proteomic profiles in synovial sepsis would increase the understanding of the molecular basis of the condition and also allow the determination of potential biomarkers for early diagnosis and treatment of the disease. We hypothesised that proteomic profile characteristics will distinguish septic from non-septic equine arthropathies and between the non-septic common arthropathies OC and OA. The objective of the study was to identify possible synovial biomarkers in equine arthropathies.

## 2 Methods

All chemicals were supplied by Sigma-Aldrich (Gillingham, UK) unless otherwise stated.

### 2.1 Sample collection

Following ethical approval and owner consent, excess aspirated SF (collected during clinical diagnostic investigations) was analysed from joints of horses presenting to The Philip Leverhulme Equine Hospital, University of Liverpool between 2014 and 2016, diagnosed with clinical OA, OC and synovial sepsis. Clinical diagnoses were determined via a combination of radiography, ultrasonography, arthroscopy and SF analysis and/or bacterial culture as previously described [11]. SF was aspirated for diagnostic purposes from the affected joints during patient examinations or at the start of surgical arthroscopy under general anaesthesia. SF was immediately placed into EDTA-containing tubes (BD Vacutainer, Belliver Industrial Estate, Plymouth, UK) and particulate and cells were removed from the SF by centrifugation (4 °C, 2540 *g* for 4 min). The cell-free supernatant was transferred to a clean uncoated 1.5 ml collection tube, snap-frozen using liquid nitrogen and stored at -80 °C. SF was collected from the distal interphalangeal, femorotibial, glenohumeral, intercarpal, metacarpophalangeal, metatarsophalangeal, patellofemoral and tarsocrural joints.

Eight healthy synovial fluid (SF) samples were obtained from the metacarpophalangeal joint of mixed breeds of horses from an abattoir with no gross joint changes, with a total score of 0 according to the Mcilwraith et al. gross scoring system, scored by two independent assessors [12]. Samples were collected as a by-product of the agricultural industry and processed within 12 h of euthanasia. The Animals (Scientific Procedures) Act 1986, Schedule 2, does not define collection from these sources as scientific procedures and ethical approval was therefore not required. Post-mortem SF samples were processed using identical protocols as for clinically collected hospital samples. Final groups consisted of OA (*n* = 4), OC (*n* = 8), synovial sepsis (*n* = 7) and healthy (n = 8) (full details in Supplementary File 1).

### 2.2 Synovial fluid preparation

SF was thawed and treated with 1 μg/ml hyaluronidase as previously described [9] and centrifuged at 10000 *g* for 10 min to remove any particulates. Protein concentrations of SF were determined by Bradford assay (Thermo Scientific, USA). A volume of sample equivalent to 5 mg of protein was added to 10 μl of beads [9]. SF samples were enriched for low abundance proteins using ProteoMiner™ beads (BioRad, UK) according to the manufacturer's instructions. Beads were re-suspended in 80 μl of 25 mM ammonium bicarbonate and 5 μl of 1% (*w*/*v*) Rapigest SF (Waters, UK) added and the sample heated at 80 °C for 10 min. On bead trypsin digestion was undertaken [9].

### 2.3 Liquid chromatography tandem mass spectrometry (LC-MS/MS) and label-free quantification

Tryptic digests (samples were diluted 5-fold in 0.1% (*v*/v) TFA/3% (v/v) acetonitrile and 2 μl loaded) were subjected to LC-MS/MS, using a 2 h gradient. Data-dependent LC-MS/MS analyses were conducted on a QExactive HF quadrupole-Orbitrap mass spectrometer (Thermo Scientific, Hemel Hempstead, UK) coupled to a Dionex Ultimate 3000 RSLC nano-liquid chromatograph (Thermo Scientific). Sample digests were loaded onto a trapping column (Acclaim PepMap 100 C18, 75 μm × 2 cm, 3 μm packing material, 100 Å) using a loading buffer of 0.1% (*v*/v) trifluoroacetic acid, 2% (v/v) acetonitrile in water for 7 min at a flow rate of 12 μl min^−1^. The trapping column was then set in-line with an analytical column (EASY-Spray PepMap RSLC C18, 75 μm × 50 cm, 2 μm packing material, 100 Å) and the peptides eluted using a linear gradient of 96.2% A (0.1% [*v*/v] formic acid):3.8% B (0.1% [v/v[formic acid in water:acetonitrile [80,20] [v/v]) to 50% A:50% B over 90 min at a flow rate of 300 nl min^−1^, followed by washing at 1% A:99% B for 5 min and re-equilibration of the column to starting conditions. The column was maintained at 40 °C, and the effluent introduced directly into the integrated nano-electrospray ionisation source operating in positive ion mode. The mass spectrometer was operated in DDA mode with survey scans between *m*/*z* 350–2000 acquired at a mass resolution of 60,000 (FWHM) at m/z 200. The maximum injection time was 100 ms, and the automatic gain control was set to 3e6. The 12 most intense precursor ions with charges states of between 2+ and 5+ were selected for MS/MS with an isolation window of 2 *m/z* units. The maximum injection time was 100 ms, and the automatic gain control was set to 1e^5^. Fragmentation of the peptides was by higher-energy collisional dissociation using normalised collision energy of 30%. Dynamic exclusion of *m/z* values to prevent repeated fragmentation of the same peptide was used with an exclusion time of 20 s.

For label-free quantification, the raw files of the acquired spectra were analysed by the ProgenesisQI™ software (Waters, Manchester, UK) [9] which aligns the files and then peak picks for quantification by peptide abundance. Briefly, the top five spectra for each feature were exported from ProgenesisQI™ and utilised for peptide identification with our local Mascot server (Version 2.6.2), searching against the Unihorse database with carbamidomethyl cysteine as a fixed modification and methionine oxidation as a variable mod, peptide mass tolerance of 10 ppm and MSMS tolerance of 0.01 Da. In this study we define differentially expressed (DE) proteins as those with a >2 fold change in expression, with a false discovery rate (FDR) adjusted *p* value of <0.05, and identified with at least two unique peptides.

Proteomic data has been deposited in the PRIDE ProteomeXchange and can be accessed using the identifier PXD011276 [13].

### 2.4 Pathway and network analysis of proteomic data in contrasts of either sepsis, OA or OC versus healthy

Network analysis, canonical pathways and upregulated regulators of proteomics data were analysed through the use of IPA (Qiagen, US). Proteins with an FDR adjusted p value of <0.05 and with 2 or more unique peptides were used for analysis, uncharacterised proteins were not included to the analysis. Gene names from the lists generated by ProgenesisQI™ were uploaded on IPA, unmapped genes were converted manually to human orthologues and as species mammals were checked for analysis.

### 2.5 Validation of mass spectrometry using ELISA

The differentially expressed proteins lactotransferrin (LTF) and insulin-like growth factor-binding protein 6 (IGFBP6) were selected to validate mass spectrometry findings as they could be measured using available enzyme linked immunosorbent assays (ELISAs) that were compatible with equine samples. Commercially available kits were used for both equine LTF (MBS902183, MyBioSource Inc., San Diego, California, USA) and equine IGFBP6 (abx574245, Abbexa Ltd., Cambridge, UK), using sandwich and competitive inhibition ELISA methodology respectively. For IGFBP6 analysis, HA treated/Costar processed native SF was diluted 1/10 whereas for LTF analysis SF was undiluted. 5–6 dependent cohort SF samples were tested per group. 100 μl aliquots of each sample were analysed in duplicate, with the absorbance measured at 450 nm and protein concentrations calculated from standard curves.

### 2.6 Additional statistical analyses

Principal component analysis (PCA) plots and heat map analysis were produced using MetaboAnalyst 4.0. The Venn diagram was produced using the online tool Venny 2.1 and box plots constructed using GraphPad Prism 8 and SPSS 24. Statistical analysis of SF protein concentration, LTF and IGFBP6 abundances were performed in Minitab version 17. These were conducted via ANOVA, using Tukey post hoc testing, with a *p* value of <0.05 considered statistically significant, following correction for multiple testing.

## 3 Results

### 3.1 Protein concentration of SF different arthropathies

**Fig. 1:**
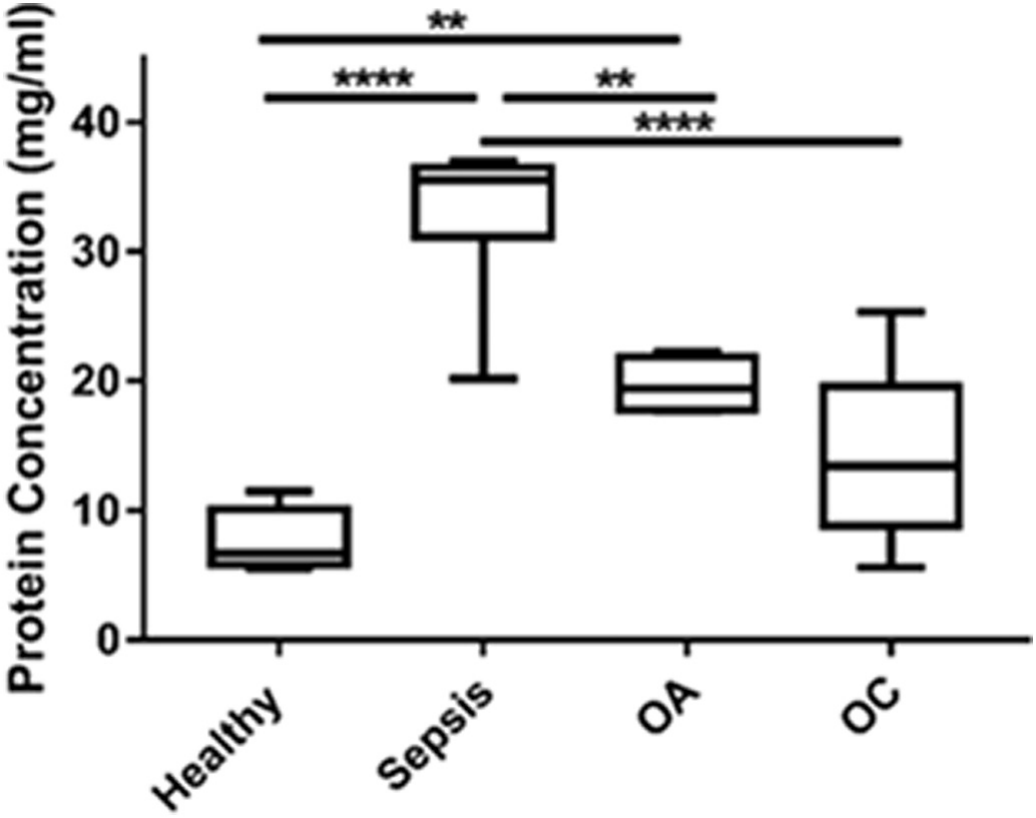
Synovial fluid protein concentrations in equine arthropathies. Protein concentrations of SF were determined by Bradford assay. Healthy (*n* = 8), OA (*n* = 4), OC (n = 8), sepsis (*n* = 7). Significant changes are demonstrated following analysis using ANOVA with Tukey's multiple comparison; ** *p* < .01, **** *p* < .0001.

The SF protein assay demonstrated that concentration was dependant on the type of arthropathy (Fig. 1).

### 3.2 Differential abundance of proteins in healthy compared to osteoarthritis, osteochondrosis and sepsis SF

**Fig. 2:**
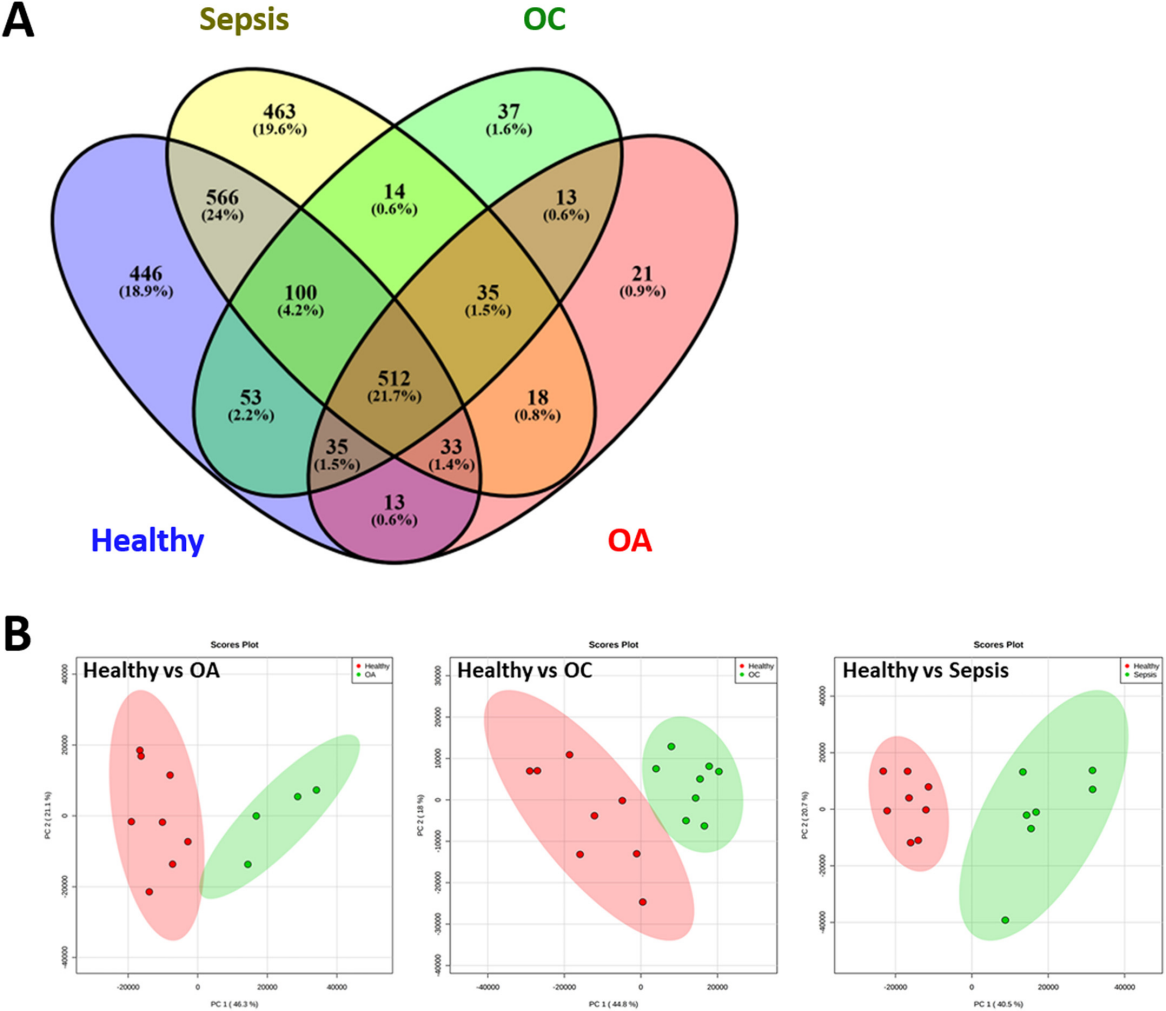
Analysis of synovial fluid proteomics. (A) Venn diagram of protein identifications for each analysed group. A minimum of 2 unique peptides were required for protein identification, with a 1% FDR correction applied. (B) Principal component analysis plots of healthy versus OA, healthy versus OC and healthy versus sepsis (FDR < 0.05, > 2-fold change and identified with at least two unique peptides).

**Fig. 3:**
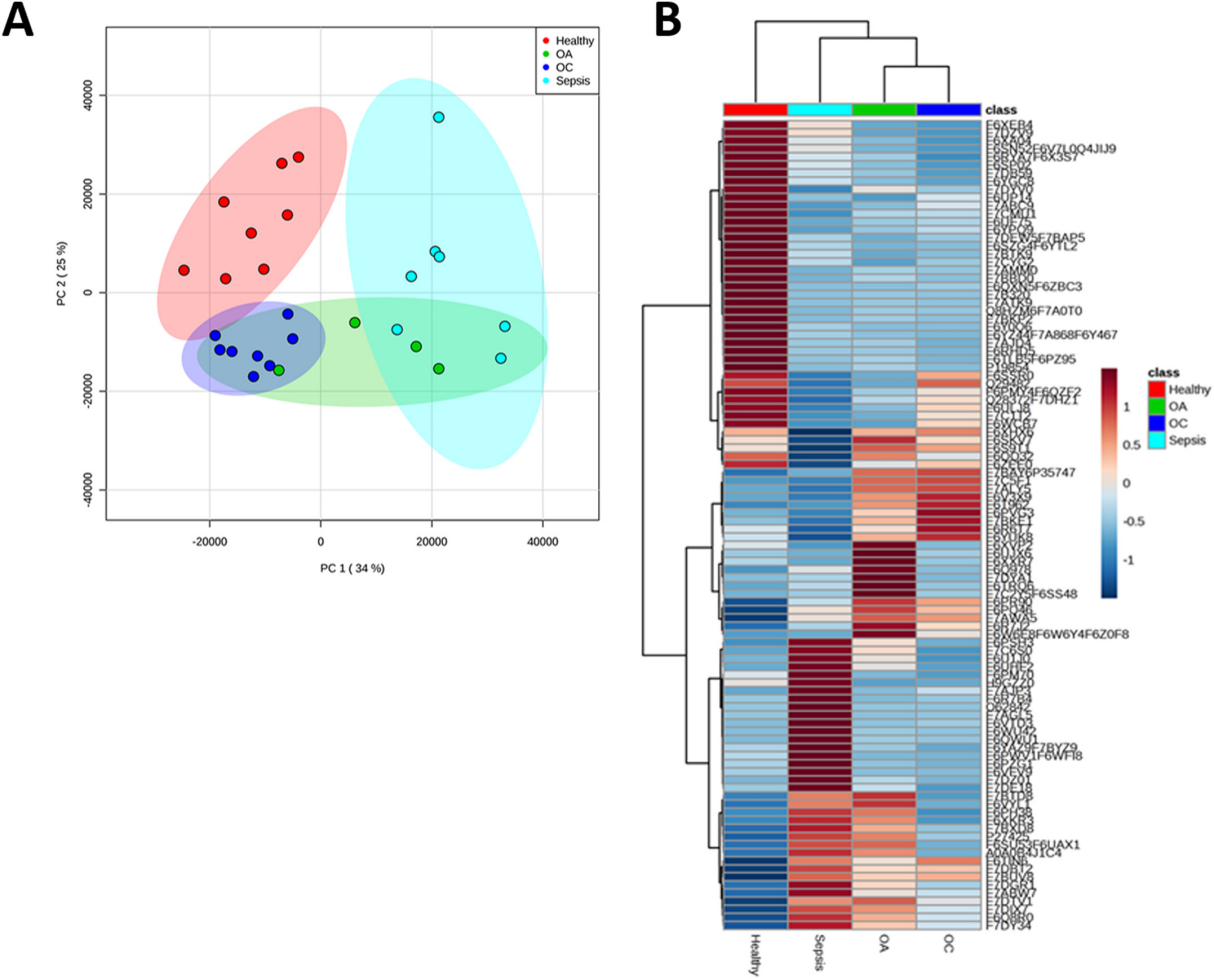
Principal component analysis and heat map analyses of healthy, OA, OC and sepsis SF samples. (A) Principal component analysis of proteins (FDR < 0.05, > 2-fold change and identified with at least two unique peptides) revealed the greatest variability was due to the type of arthropathy distinguished by shading (healthy; red, OA; green, OC; blue, sepsis; turquoise). (B) Heat maps of the top 100 differentially expressed proteins show distinguishable profiles based on arthropathy type. Each column indicates a different sample group. Each row indicates one protein. Red shading indicates increased expression and blue decreased expression. (For interpretation of the references to colour in this figure legend, the reader is referred to the web version of this article.)

From the aggregate data file produced from all samples 1385 protein families, at a 1% FDR, were identified (Supplementary File 2). ProgenesisQI™ identified 750 proteins DE when all groups were compared using a 4-way analysis (>2-fold change, FDR < 0.05 and identified with at least two unique peptides) (Supplementary File 3). The number of protein identifications in each analysed group are shown in Fig. 2A. The number of DE proteins in 2-way contrasts is shown in Table 1 (with complete protein lists shown in Supplementary File 4) and multivariate 2-way comparisons of protein profiles shown in Fig. 2B. Unsupervised multivariate analysis (Principal Component Analysis (PCA)) demonstrated clear variance between groups in the 4-way analysis (Fig. 3A).

### 3.3 DE proteins were filtered to include proteins identified with two or more peptides, q < 0.05 and > 2 fold change in expression

*Canonical pathways and network analysis of osteoarthritis, osteochondrosis and sepsis SF.*

**Table 1:** Number of proteins DE in two-way comparisons.

Contrast	Number of Proteins DE	Increased	Decreased
Healthy versus Sepsis	386	205	181
Healthy versus OA	430	331	99
Healthy versus OC	539	474	65
OA versus Sepsis	53	22	31
OC versus Sepsis	454	75	379
OA versus OC	0	0	0

**Fig. 4:**
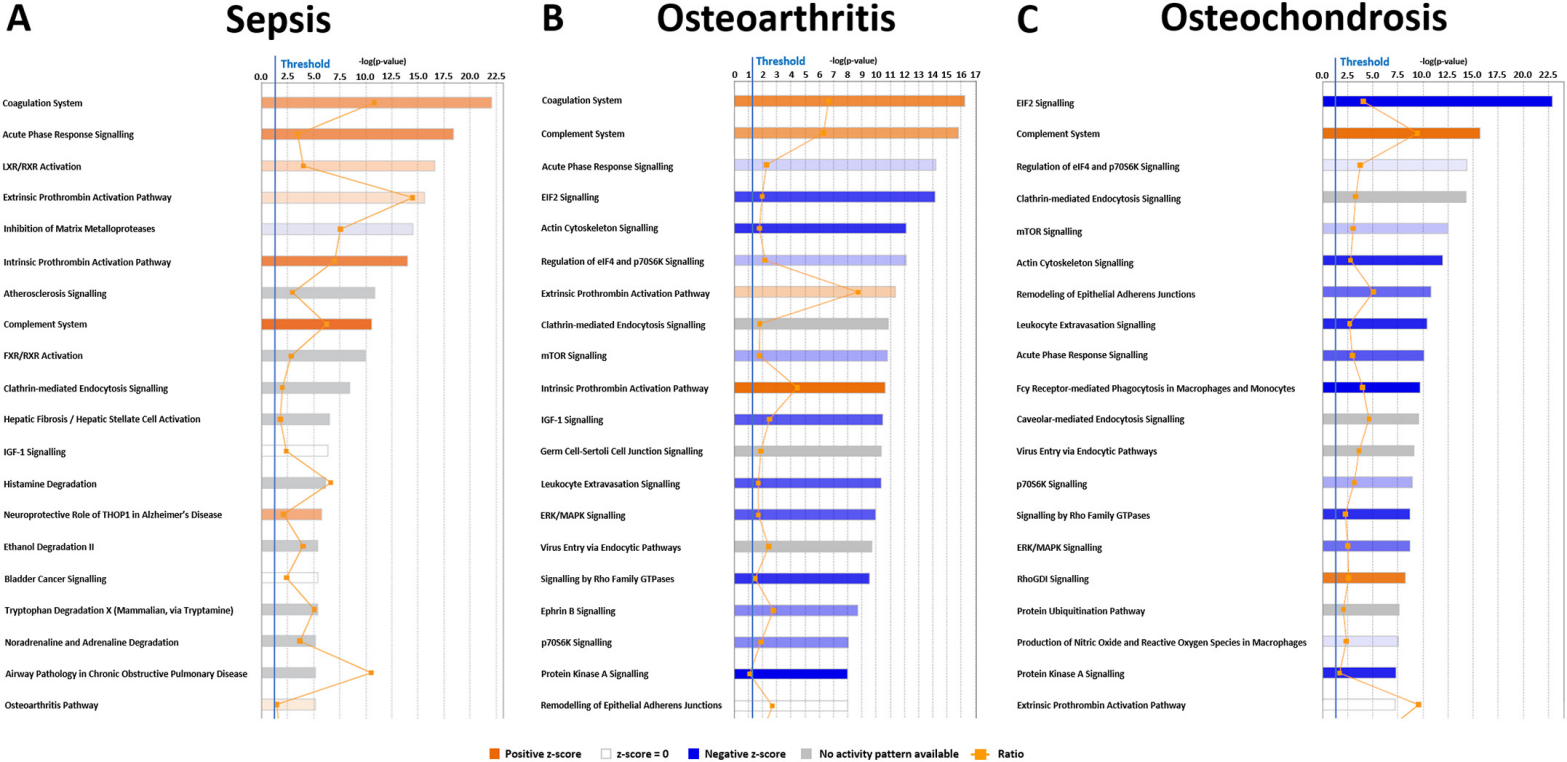
Canonical pathways of differentially abundant proteins in equine synovial fluid of (A) sepsis, (B) OA and (C) OC compared to healthy samples. Bars represent the significance of the canonical pathway, calculated by a right-sided Fisher's exact test. The tallest bars represent the canonical pathways that are the least likely to have been identified due to random chance. Upregulated canonical pathways are shown in orange and downregulated pathways in blue. (For interpretation of the references to colour in this figure legend, the reader is referred to the web version of this article.)

Canonical pathway analysis was undertaken on differentially abundant proteins, contrasting either OA, OC or sepsis, versus healthy (Supplementary File 5). When sepsis samples were compared to healthy it was revealed that the predicted upregulation of coagulation system (*p* = 7.94 × 10^−23^), acute phase response signalling (*p* = 3.98 × 10^−19^), LXR/RXR activation (*p* = 2.51 × 10^−17^), extrinsic and intrinsic prothrombin activation pathways (p = 2 × 10^−16^ and *p* = 1 × 10^−14^), complement system (*p* = 3.16 × 10^−11^), neuroprotective role of THOP1 in Alzheimer's disease (*p* = 1.55 × 10^−6^) and OA pathway (*p* = 7.08 × 10^−6^); and inhibition of matrix metalloproteases was downregulated (p = 2.51 × 10–^15^) (Fig. 4A). Whereas pathway analysis of OA versus healthy demonstrated upregulation of the coagulation system (*p* = 5.01 × 10^−17^), complement system (*p* = 1.58 × 10^−16^), extrinsic and intrinsic prothrombin activation pathways (p = 5.01 × 10^−12^ and p = 2 × 10^−11^) (Fig. 4B). Comparison of differential abundant proteins in OC compared to healthy samples demonstrated an upregulation in complement system (p = 2 × 10^−16^) and RhoGDI signalling (*p* = 5.62 × 10^−9^) (Fig. 4C). Downregulated canonical pathways of OA or OC versus healthy had a substantial overlap including acute phase response (OA *p* = 6.31 × 10^−15^, OC *p* = 7.94 × 10^−11^), EIF2 signalling (OA p = 7.94 × 10^−15^, OC *p* = 1.26 × 10^−23^), actin cytoskeleton signalling (OA p = 7.94 × 10^−13^, OC p = 1 × 10^−12^), regulation of eIF4 and p7056K signalling (OA p = 7.94 × 10^−13^, OC *p* = 3.98 × 10^−15^), mTOR signalling (OA p = 1.58 × 10^−11^, OC *p* = 3.16 × 10–^13^), leukocyte extravasation signalling (OA p = 3.98 × 10^−11^, OC p = 3.98 × 10^−11^), signalling by Rho family GTPases (OA *p* = 2.82 × 10^−10^, OC *p* = 1.82 × 10^−9^), p7056K signalling (OA *p* = 8.91 × 10^−9^, OC *p* = 1.15 × 10^−9^) and protein kinase A signalling (OA *p* = 1.02 × 10^−8^, OC *p* = 4.9 × 10^−8^). IGF-1 signalling (p = 3.16 × 10^−11^) and ephrin B signalling (*p* = 1.91 × 10^−9^) were downregulated pathways in OA only; and Fcy receptor-moderated phagocytosis in macrophages and monocytes (p = 1.91 × 10^−10^), production of nitric oxide and reactive oxygen species in macrophages (*p* = 2.88 × 10^−8^) present in OC only.

**Fig. 5:**
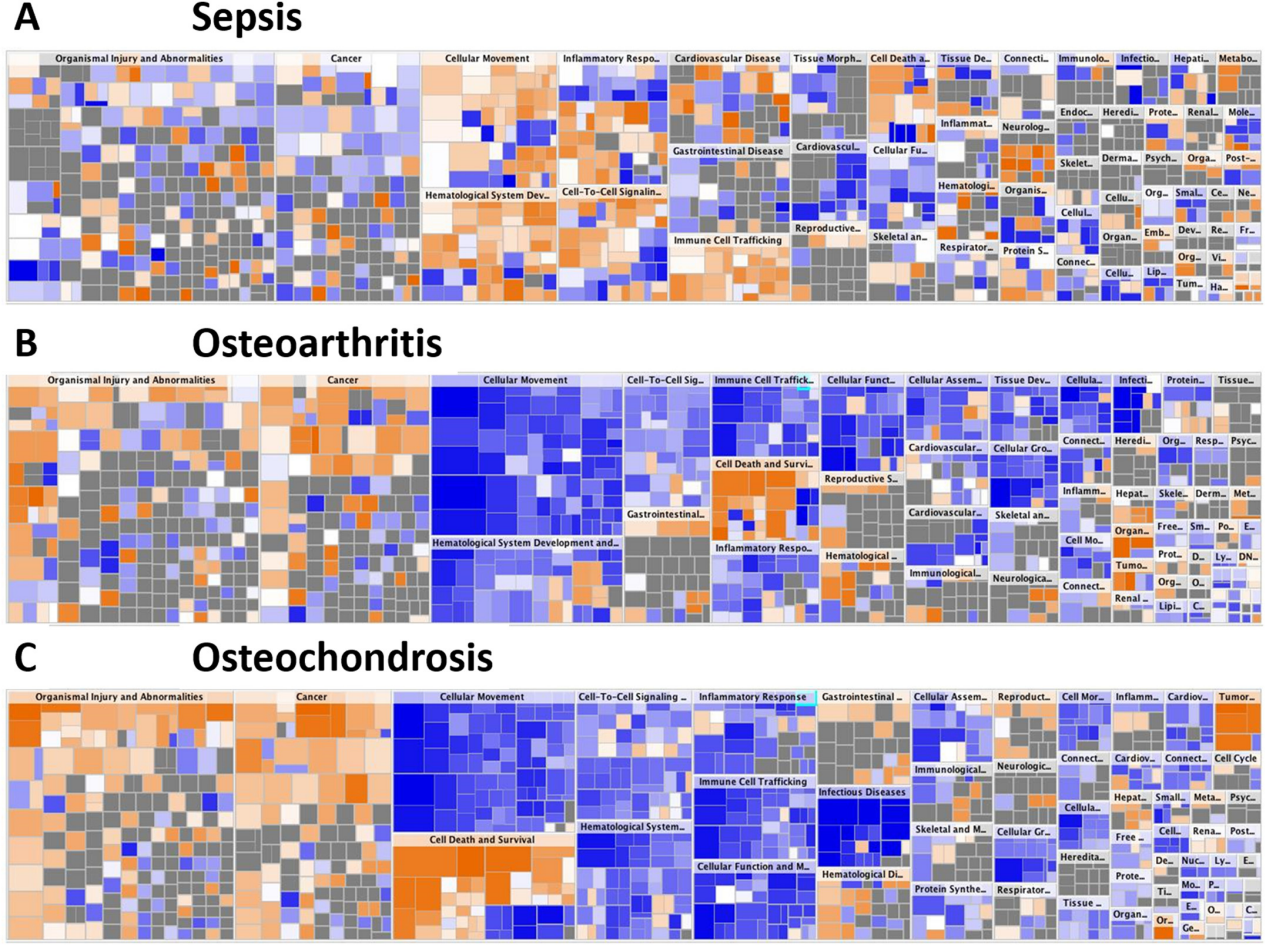
Heat map identifying canonical pathway groupings for molecular and cellular functions altered in equine synovial fluid of (A) sepsis, (B) OA and (C) OC in comparison to healthy samples. Squares are coloured according to their Z score, with orange upregulated in the disease state and blue downregulated, with the colour intensity indicating prediction strength. The Z score represents whether the up- or downregulation of the proteins within that function will lead to activation (positive Z score) or inhibition (negative Z score) of the function. (For interpretation of the references to colour in this figure legend, the reader is referred to the web version of this article.)

**Fig. 6:**
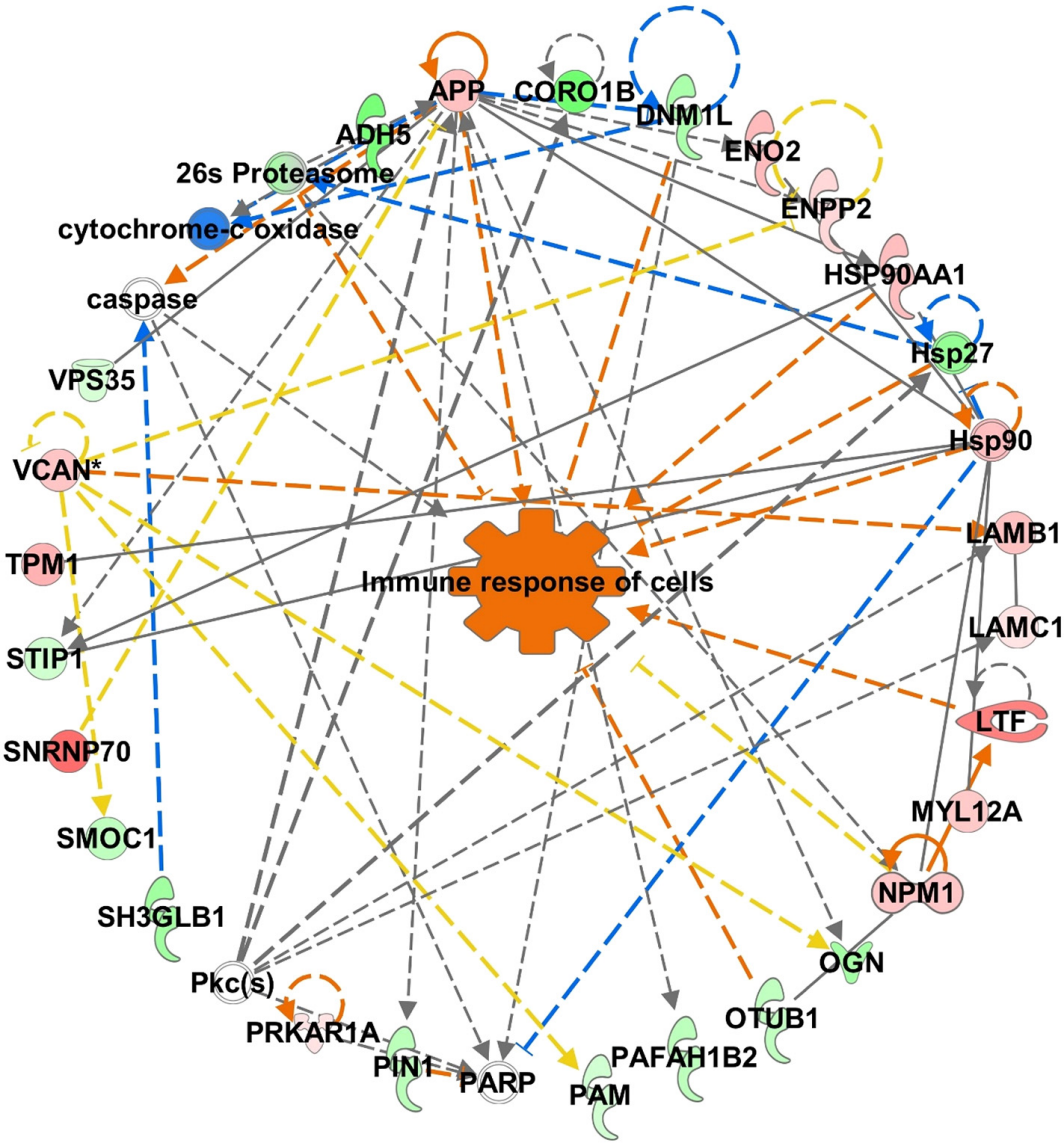
Network involved in the immune response of cells mapped from the differential abundant proteins of septic equine synovial fluid. Red nodes represent greater protein abundance in sepsis; green nodes represent lower protein abundance in sepsis; and white nodes represent molecules mapped to this network and not present in the dataset. Orange arrows represent predicted activation, blue arrows predicted inhibition and yellow arrows indicate results inconsistent with the state of downstream molecules. (For interpretation of the references to colour in this figure legend, the reader is referred to the web version of this article.)

Compared to healthy SF, in sepsis there was an upregulation in cellular movement, haematological system development, inflammatory response, cell-to-cell signalling and immune cell trafficking and this was in distinct contrast to these pathways in OA and OC in which they were downregulated (Fig. 5A). Similarities between groupings of canonical pathways in OA (Fig. 5B) and OC (Fig. 5C) were identified. The groups of organismal injury and abnormalities, and cancer had the highest overlap in healthy versus sepsis/OA/OC, however the activation of most pathways could not be predicted by IPA, with an overall trend to be downregulated. Organismal injury and cancer had the most overlap in healthy versus OA or OC as well. These were predicted to be upregulated in OA compared to sepsis and even more upregulated in OC. (See Fig. 6.)

*ELISA validation of lactotransferrin and Insulin-like growth factor-binding protein 6.*

**Fig. 7:**
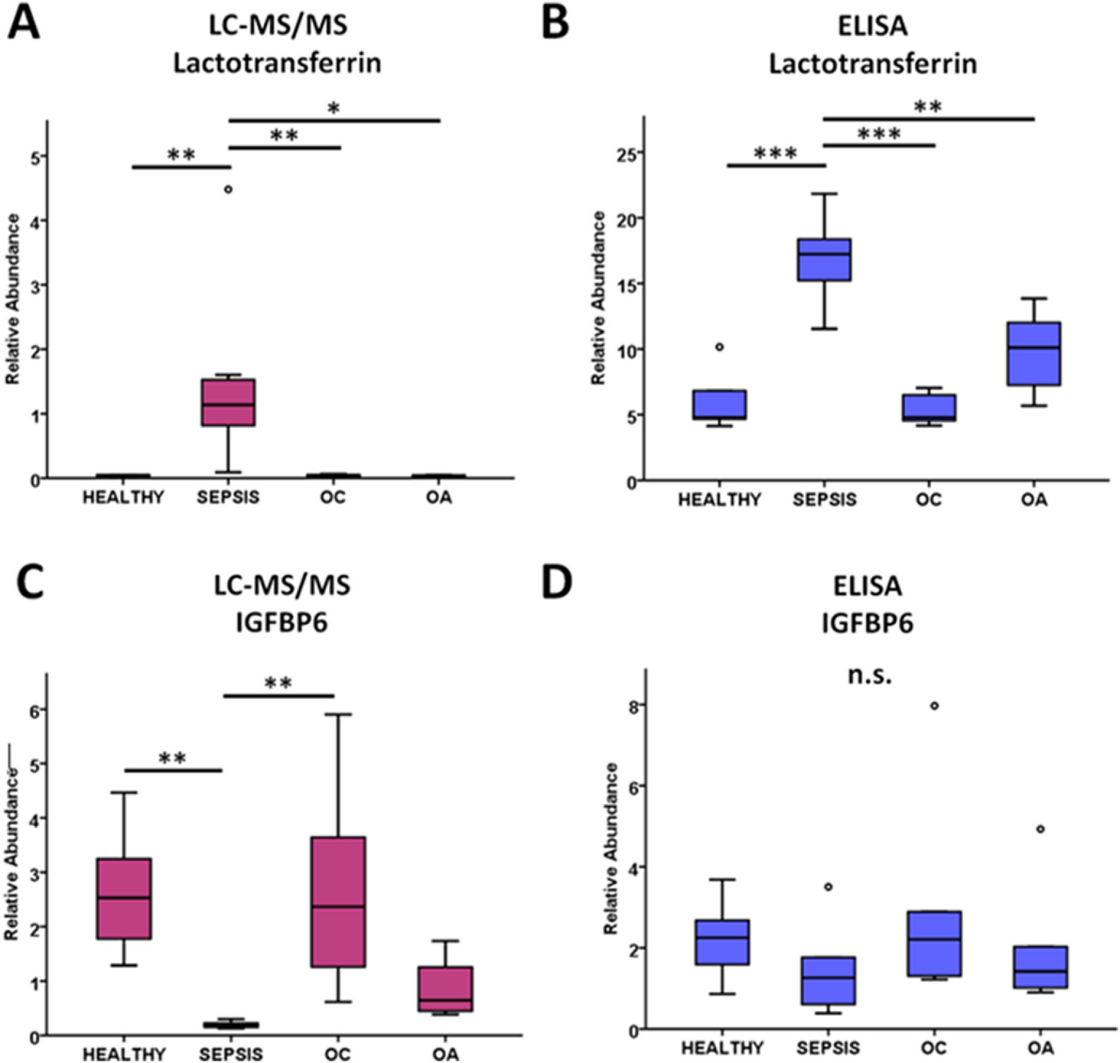
ELISA validation of LC-MS/MS results for lactotransferrin (A and B) and insulin-like growth factor binding protein 6 (IGFBP6) (C and D). IGFBP6 normalised to total protein. ELISA: *n* = 5–6/group. * = *p* < .05, ** = *p* < .01, *** = *p* < .001. OA = osteoarthritis, OC = osteochondrosis, n.s. = non-significant.

LTF ELISA assay results corroborated mass spectrometry findings with a statistically elevated abundance present within sepsis SF samples compared to all other groups (Fig. 7). IGFBP6 ELISA results produced a similar trend to that identified via mass spectrometry; however these results were not of statistical significance.

## 4 Discussion

Articular pathologies have an important clinical relevance in horses; however, to date the veterinary practitioner can find it difficult to achieve a timely diagnosis and prognostication of these conditions in horses affected by joint pathologies. This study has confirmed that SF protein concentration is associated with the type of arthropathy and has identified pathways and possible markers that may enable further understanding, together with rapid and accurate identification of these conditions in horses and potentially other species. For the first time the study of the proteomic profile of SF has identified potential SF markers of synovial sepsis. The proteomic profile can discriminate between septic and non-septic articular pathologies, and between non-septic conditions such as OC and OA. The implications highlighted by protein changes attributable to different pathways has increased our knowledge on articular pathophysiology.

Within this study, elevated synovial levels of lactotransferrin (LTF) were identified within the sepsis group compared to the other pathologies. LTF, also known as lactoferrin, is an 80 kDa multifunctional glycoprotein, abundant in various biofluids, primarily transporting iron ions, but also exhibiting anti-microbial, anti-inflammatory, and immune modulatory effects [[14], [15], [16]] LTF provides anti-bacterial action against various bacterial species, inhibiting the growth of *Staphylococcus epidermidis*, antibiotic-resistant *Klebseilla* and *Staphylococcus aureus* in vitro and reducing blood and hepatic infection severity following *Escherichia coli* enteral infection in rats [[17], [18], [19], [20]] LTF is present within specific granules of neutrophils with elevations in LTF levels potentially representing a marker of neutrophil granulocyte activation [21]. LTF levels within human SF have been found to be closely correlated to the level of SF neutrophilia, indicating the degree of inflammatory response [22]. Various studies on SF from RA patients have identified increased synovial levels of LTF [[23], [24], [25]]. LTF has been found to activate expression of bone morphogenetic protein 7 (BMP7) within porcine articular chondrocytes, through the mitogen-activated protein kinase ERK pathway [26]. As BMP7 has an important role in maintaining homeostasis of articular cartilage, the authors proposed LTF exhibits a protective function following inflammation within the joint. During synovial sepsis, there is an upregulation of collagen catabolism due to an increased cytokine concentration leading to the release of matrix metalloproteinases [27]. Thus, following synovial sepsis the release of LTF from neutrophil granulocytes may have a multi-functional role, both providing a direct antibacterial function and enabling a degree of chondroprotection. Clinically, LTF diet supplementation of neonates has demonstrated an ability to reduce the occurrence of late onset sepsis [19,[28], [29], [30]]. However, elevations in synovial LTF levels have not previously been identified as a marker of synovial sepsis.

In this study, reduced SF IGFBP6 was identified within the sepsis group compared to other joint pathologies. This protein is involved in cartilage and bone homeostasis but is also expressed in the synovium, particularly in RA versus OA [31]. IGFBP6 is a 30 kDa protein which is secreted into extracellular regions where it can subsequently interact with insulin growth factors (IGFs). IGFBP6, unlike other IGFBPs, binds to IGF2 with higher affinity than IGF1, inhibiting IGF2 activity [32]. IGFBP6 has previously been identified in human SF, with synovial levels decreased in RA compared to OA [31]. Postoperative sepsis significantly alters the growth hormone/IGF axis, reducing GH-dependent molecule secretion (i.e. IGFBP3) and increasing GH-independent molecule secretion (i.e. IGFBP6) [33]. However, decreased levels of synovial IGFBP6 have not previously been identified for synovial sepsis in horses. IGFBPs are known regulators of insulin resistance, with increased serum IGFBP6 levels identified within type 1 diabetes, and thus high systemic glucose [34]. Reduced synovial glucose concentrations is a known clinical parameter associated with synovial sepsis [35]. Thus, the localised reduced abundance of IGFBP6 may be reflective or the decreased synovial glucose levels.

Protein differences were greatest between synovial sepsis and other groups probably reflecting the more distant pathological pathways implicated. Pathway analysis of DE proteins in sepsis compared to healthy revealed upregulation of a number of canonical pathways including the coagulation system, acute phase response signalling and complement system. Systemic inflammation results in activation of coagulation, due to tissue factor-mediated thrombin generation, downregulation of physiological anticoagulant mechanisms, and inhibition of fibrinolysis [36]. In healthy SF, coagulation proteins have a low abundance with the elevated SF levels identified within sepsis in our study probably due to thrombin activation and coagulation, also reported in inflamed human joints [37]. In an equine model of joint inflammation changes in fibrinogen and thrombin/antithrombin have also been reported [38]. The upregulation in sepsis of the acute phase response signalling pathway is supports findings of our group and others that acute phase proteins such as serum amyloid A increase in equine joint sepsis [11,39].

The upregulation of the complement system in joint sepsis is not unexpected. The complement system is one of the key players in the defence against infections [40]. Its activation during the innate immune response leads to the generation of several proteins that contribute to the lysis and opsonisation of microorganisms, regulate inflammatory reactions and bridge innate immunity with the subsequent adaptive immune response. Depletion in the complement system in animal models increases the severity of septic arthritis [41].

Interestingly eukaryotic translation initiation factors (EIF2) and mTOR signalling were downregulated pathways in both OA and OC compared to healthy SF. OC is a juvenile osteoarticular disorder affecting several mammalian species. In horses, it is a multifactorial disease with focal disruption of endochondral ossification leading to the development of osteoarticular lesions [42]. Nevertheless, OC pathophysiology is poorly understood. Protein synthesis requires cooperation among a large number of polypeptides including ribosomal proteins, modification enzymes and ribosome associated translation factors. The initiation phase of protein synthesis requires a set of EIFs. A reduction in these proteins in OA and OC is interesting as in both conditions there is an imbalance between cartilage protein anabolism and catabolism leading to a net loss of cartilage. However there is an increase in mTOR in end-stage OA and mTOR is important in maintaining cartilage homeostasis [43]. Furthermore signalling through mTOR is an important link between synovitis and structural damage in inflammatory arthritis [44]. In OA and OC proteins in the SF in our study may be derived from cartilage, subchondral bone or synovium. Further studies are required to determine the relevance of these changes at the SF proteome level to the pathogenesis of these diseases since mTOR inhibition protects cartilage from experimental OA (42).

Using IPA we have identified a number of proteins requiring further investigation as potential biomarkers of disease and/or therapeutic targets (Supplementary File 5). For example, Nuclear factor (erythroid-derived 2) (NFE2L2) was identified as the most significantly inhibited upstream regulator in OC. Interestingly although also identified as a potential upstream inhibitory regulator in OA the effect in OC was much more significant. This master regulator of redox homeostasis regulates the expression of antioxidant proteins that protect against oxidative damage triggered by injury and inflammation. It has a potentially protective role in joint inflammation and degeneration [45]. We have therefore identified a catalogue of proteins for future studies.

Healthy SF samples were collected following euthanasia whilst samples from the pathological groups were predominantly collected from live horses during clinical examination. Thus, it cannot be ruled out that differentially abundant proteins identified within the healthy group may be resultant of post mortem change. However, post mortem processing took place within 12 h of euthanasia and therefore the impact on the synovial proteome is likely to be minimal. Due to the limited sample sizes included within this study, SF aspirated from various joints was included. However, despite the inclusion of analysis of SF from multiple sites, the clear separation identified in the proteomic profiles of different joint diseases strengthens the conclusion that the differentially expressed proteins are resultant of the joint pathology opposed to joint location. Additionally, horses with different pathologies investigated within this study may experience changes in management, including diet and exercise, which may subsequently influence synovial protein composition. However, these systemic effects are likely to have a limited effect on the synovial proteome compared to the more significant impact of local joint pathology.

Within this study, ELISA validations of LTF and IGFBP6 synovial abundances were undertaken on the same samples as those used for mass spectrometry analysis due to limited availability of additional SF samples. Thus, to confirm the results of this study identifying these as potential synovial sepsis markers, further validation needs to be undertaken using an independent cohort before these proteins are investigated further.

## 5 Conclusion

This study has identified that the equine SF proteome exhibits distinctive profile changes between OA, OC, synovial sepsis and healthy joints. Elevated synovial abundance of LTF and decreased IGFBP6 were both found to distinguish synovial sepsis from all other study groups. Thus, these protein markers may have a future role in clinical practice to enable an earlier and reliable diagnosis of synovial sepsis.

## Appendix A Supplementary data

Supplementary File 1 Donor details. Image 1

Supplementary File 2 Aggregate data file produced from all samples 1385 protein families at a 1% false discovery rate (FDR) Image 2

Supplementary File 3 Proteins differentially expressed in the four way analysis Image 3

Supplementary File 4 Proteins differentially expressed in each two way analysis Image 4

Supplementary File 5 Pathway analysis results using IPA Image 5

## References

[1] Rubio-Martinez L.M., Elmas C.R., Black B., Monteith G. (2012). Clinical use of antimicrobial regional limb perfusion in horses: 174 cases (1999-2009). J. Am. Vet. Med. Assoc..

[2] Dicker K.T., Gurski L.A., Pradhan-Bhatt S., Witt R.L., Farach-Carson M.C., Jia X. (2014). Hyaluronan: a simple polysaccharide with diverse biological functions. Acta Biomater..

[3] Ruiz-Romero C., Blanco F.J. (2010). Proteomics role in the search for improved diagnosis, prognosis and treatment of osteoarthritis. Osteoarthr. Cartil..

[4] Mateos J., Lourido L., Fernandez-Puente P., Calamia V., Fernandez-Lopez C., Oreiro N. (2012). Differential protein profiling of synovial fluid from rheumatoid arthritis and osteoarthritis patients using LC-MALDI TOF/TOF. J. Proteome.

[5] Frisbie D.D., Stick JAAaJA (2006). Synovial joint biology and pathobiology. Equine Surgery.

[6] Sanchez-Teran A.F., Bracamonte J.L., Hendrick S., Riddell L., Musil K., Hoff B. (2016). Effect of repeated through-and-through joint lavage on serum amyloid a in synovial fluid from healthy horses. Vet. J..

[7] Jacobsen S., Thomsen M.H., Nanni S. (2006). Concentrations of serum amyloid a in serum and synovial fluid from healthy horses and horses with joint disease. Am. J. Vet. Res..

[8] Sinovich M., Villarino N.F., Singer E.R., Robinson C.S., Rubio-Martinez L.M. (2018). Investiagtion of blood serum amyloid a concentrations in horses to differentiate synovial sepsis from inflammation and determine prognosis an response to treatment. Vet. Surg..

[9] Peffers M.J., McDermott B., Clegg P.D., Riggs C.M. (2015). Comprehensive protein profiling of synovial fluid in osteoarthritis following protein equalization. Osteoarthr. Cartil..

[10] Jacobsen S., Niewold T.A., Halling-Thomsen M., Nanni S., Olsen E., Lindegaard C. (2006). Serum amyloid a isoforms in serum and synovial fluid in horses with lipopolysaccharide-induced arthritis. Vet. Immunol. Immunopathol..

[11] Robinson C.S., Singer E.R., Piviani M., Rubio-Martinez L.M. (2017). Are serum amyloid A or D-lactate useful to diagnose synovial contamination or sepsis in horses?. Vet. Rec..

[12] McIlwraith C.W., Frisbie D.D., Kawcak C.E., Fuller C.J., Hurtig M., Cruz A. (2010). The OARSI histopathology initiative - recommendations for histological assessments of osteoarthritis in the horse. Osteoarthr. Cartil..

[13] Vizcaino J.A., Csordas A., del-Toro N., Dianes J.A., Griss J., Lavidas I. (2016). 2016 update of the PRIDE database and its related tools. Nucleic Acids Res..

[14] Albar A.H., Almehdar H.A., Uversky V.N., Redwan E.M. (2014). Structural heterogeneity and multifunctionality of lactoferrin. Curr. Protein Pept. Sci..

[15] Paramasivam M., Saravanan K., Uma K., Sharma S., Singh T.P., Srinivasan A. (2002). Expression, purification, and characterization of equine lactoferrin in Pichia pastoris. Protein Expr. Purif..

[16] Ye Q., Zheng Y., Fan S., Qin Z., Li N., Tang A. (2014). Lactoferrin deficiency promotes colitis-associated colorectal dysplasia in mice. PLoS One.

[17] Edde L., Hipolito R.B., Hwang F.F., Headon D.R., Shalwitz R.A., Sherman M.P. (2001). Lactoferrin protects neonatal rats from gut-related systemic infection. Am. J. Physiol. Gastrointest. Liver Physiol..

[18] Nibbering P.H., Ravensbergen E., Welling M.M., van Berkel L.A., van Berkel P.H., Pauwels E.K. (2001). Human lactoferrin and peptides derived from its N terminus are highly effective against infections with antibiotic-resistant bacteria. Infect. Immun..

[19] Pammi M., Abrams S.A. (2015). Oral lactoferrin for the prevention of sepsis and necrotizing enterocolitis in preterm infants. Cochrane Database Syst. Rev..

[20] Valenti P., Antonini G. (2005). Lactoferrin: an important host defence against microbial and viral attack. Cell. Mol. Life Sci..

[21] Afeltra A., Caccavo D., Ferri G.M., Addessi M.A., De Rosa F.G., Amoroso A. (1997). Expression of lactoferrin on human granulocytes: analysis with polyclonal and monoclonal antibodies. Clin. Exp. Immunol..

[22] Bennett R.M., Skosey J.L. (1977). Lactoferrin and lysozyme levels in synovial fluid: differential indices of articular inflammation and degradation. Arthritis Rheum..

[23] Abbink J.J., Kamp A.M., Nieuwenhuys E.J., Nuijens J.H., Swaak A.J., Hack C.E. (1991). Predominant role of neutrophils in the inactivation of alpha 2-macroglobulin in arthritic joints. Arthritis Rheum..

[24] Caccavo D., Garzia P., Sebastiani G.D., Ferri G.M., Galluzzo S., Vadacca M. (2003). Expression of lactoferrin on neutrophil granulocytes from synovial fluid and peripheral blood of patients with rheumatoid arthritis. J. Rheumatol..

[25] Decoteau E, Yurchak AM, Partridge RE, Tomasi TB, Jr. Lactoferrin in synovial fluid of patients with inflammatory arthritis. Arthritis Rheum. 1972;15:324–5. 10.1002/art.1780150317 4555646

[26] Zhang C., Li Y., Tang W., Kamiya N., Kim H. (2013). Lactoferrin activates BMP7 gene expression through the mitogen-activated protein kinase ERK pathway in articular cartilage. Biochem. Biophys. Res. Commun..

[27] Shirtliff M.E., Mader J.T. (2002). Acute septic arthritis. Clin. Microbiol. Rev..

[28] Baveye S., Elass E., Mazurier J., Spik G., Legrand D. (1999). Lactoferrin: a multifunctional glycoprotein involved in the modulation of the inflammatory process. Clin. Chem. Lab. Med..

[29] Baynes R.D., Bezwoda W.R. (1994). Lactoferrin and the inflammatory response. Adv. Exp. Med. Biol..

[30] Kruzel M.L., Harari Y., Mailman D., Actor J.K., Zimecki M. (2002). Differential effects of prophylactic, concurrent and therapeutic lactoferrin treatment on LPS-induced inflammatory responses in mice. Clin. Exp. Immunol..

[31] Alunno A., Bistoni O., Manetti M., Cafaro G., Valentini V., Bartoloni E. (2017). Insulin-like growth factor binding protein 6 in rheumatoid arthritis: a possible novel chemotactic factor?. Front. Immunol..

[32] Aboalola D., Han V.K.M. (2017). Different effects of insulin-like growth Factor-1 and insulin-like growth Factor-2 on myogenic differentiation of human mesenchymal stem cells. Stem Cells Int..

[33] Nedic O., Robajac D., Sunderic M., Miljus G., Dukanovic B., Malenkovic V. (2013). Detection and identification of oxidized insulin-like growth factor-binding proteins and receptors in patients with colorectal carcinoma. Free Radic. Biol. Med..

[34] Lu S., Purohit S., Sharma A., Zhi W., He M., Wang Y. (2012). Serum insulin-like growth factor binding protein 6 (IGFBP6) is increased in patients with type 1 diabetes and its complications. Int. J. Clin. Exp. Med..

[35] Anderson J.R., Phelan M.M., Clegg P.D., Peffers M.J., Rubio-Martinez L.M. (2018). Synovial fluid metabolites differentiate between septic and nonseptic joint pathologies. J. Proteome Res..

[36] Levi M., Keller T.T., van Gorp E., ten Cate H. (2003). Infection and inflammation and the coagulation system. Cardiovasc. Res..

[37] So A.K., Varisco P.A., Kemkes-Matthes B., Herkenne-Morard C., Chobaz-Peclat V., Gerster J.C. (2003). Arthritis is linked to local and systemic activation of coagulation and fibrinolysis pathways. J. Thromb. Haemost..

[38] Andreassen S.M., Vinther A.M.L., Nielsen S.S., Andersen P.H., Tnibar A., Kristensen A.T. (2017). Changes in concentrations of haemostatic and inflammatory biomarkers in synovial fluid after intra-articular injection of lipopolysaccharide in horses. BMC Vet. Res..

[39] Jacobsen S., Kjelgaard-Hansen M. (2008). Evaluation of a commercially available apparatus for measuring the acute phase protein serum amyloid a in horses. Vet. Rec..

[40] Blom A.M. (2017). The role of complement inhibitors beyond controlling inflammation. J. Intern. Med..

[41] Sakiniene E., Bremell T., Tarkowski A. (1999). Complement depletion aggravates Staphylococcus aureus septicaemia and septic arthritis. Clin. Exp. Immunol..

[42] Olsson S.E., Reiland S. (1978). The nature of osteochondrosis in animals. Summary and conclusions with comparative aspects on osteochondritis dissecans in man. Acta Radiol. Suppl..

[43] Zhang Y., Vasheghani F., Li Y.H., Blati M., Simeone K., Fahmi H. (2015). Cartilage-specific deletion of mTOR upregulates autophagy and protects mice from osteoarthritis. Ann. Rheum. Dis..

[44] Cejka D., Hayer S., Niederreiter B., Sieghart W., Fuereder T., Zwerina J. (2010). Mammalian target of rapamycin signaling is crucial for joint destruction in experimental arthritis and is activated in osteoclasts from patients with rheumatoid arthritis. Arthritis Rheum..

[45] Maicas N., Ferrandiz M.L., Brines R., Ibanez L., Cuadrado A., Koenders M.I. (2011). Deficiency of Nrf2 accelerates the effector phase of arthritis and aggravates joint disease. Antioxid. Redox Signal..

[46] Chiaradia E., Pepe M., Tartaglia M., Scoppetta F., D'Ambrosio C., Renzone G., Avellini L., Moriconi F., Gaiti A., Bertuglia A., Beccati F., Scaloni A. (2012). Gambling on putative biomarkers of osteoarthritis and osteochondrosis by equine synovial fluid proteomics. J. Proteomics.

